# Human metapneumovirus: understanding the molecular mechanisms and pathology of infection

**DOI:** 10.1128/jvi.00284-25

**Published:** 2025-09-22

**Authors:** Chase J. Heim, Bernadette G. van den Hoogen, Rebecca E. Dutch

**Affiliations:** 1 Department of Molecular and Cellular Biochemistry, University of Kentucky, College of Medicine 12252, Lexington, Kentucky, USA; 2 Department of Viroscience, Erasmus Medical Center 6993, Rotterdam, the Netherlands; Indiana University Bloomington, Bloomington, Indiana, USA

**Keywords:** pneumovirus, HMPV

## Abstract

Human metapneumovirus (HMPV) is a common and globally prevalent respiratory virus that can cause clinical symptoms ranging from mild respiratory illness to severe bronchiolitis and pneumonia, with substantial morbidity and mortality. HMPV accounts for a substantial health care and economic burden, with high hospitalization rates. Consequently, there is an urgent need for effective preventive and therapeutic interventions. The development of these interventions requires comprehensive knowledge of the virus’s biology, including characteristics, epidemiology, evolution, virus-host interactions, and host immune responses. Despite being discovered nearly 25 years ago, HMPV has remained relatively underrecognized, resulting in limited awareness of its true impact and delays in the development of treatment options. Recent studies have demonstrated the emergence of novel genotypes and provided more insight into viral replication, spread, and host immune responses. In this review, we highlight the clinical significance of HMPV and explore the molecular mechanisms the virus employs throughout the course of an infection.

## INTRODUCTION

Human metapneumovirus (HMPV) was first identified in 2001 by researchers who analyzed samples from 28 patients suffering from respiratory tract illnesses (1). Since its identification, HMPV has been found to be globally prevalent (2, 3), with most individuals experiencing their first infection by the age of 5 years (4). Despite being discovered in 2001, serological studies with sera collected in 1958 demonstrated that HMPV had been circulating since the 1950s (1). Later on, phylogenetic analysis suggested that HMPV evolved from avian metapneumovirus after a zoonotic event at least 200 years ago (5, 6).

The symptoms of HMPV infection can range from mild respiratory tract illness to severe bronchiolitis and pneumonia (1). The majority of HMPV infections are mild; however, vulnerable groups such as children, older adults, and the immunocompromised are at a much higher risk of developing severe symptoms. In 2003, a two-season study analyzed samples of hospitalized individuals with respiratory infections and showed that 7% of patients were positive for HMPV (4). Numerous other studies have shown a range of incidence rates for HMPV-positive hospitalizations, which vary based on the target population and detection method used.

**Fig 1:**
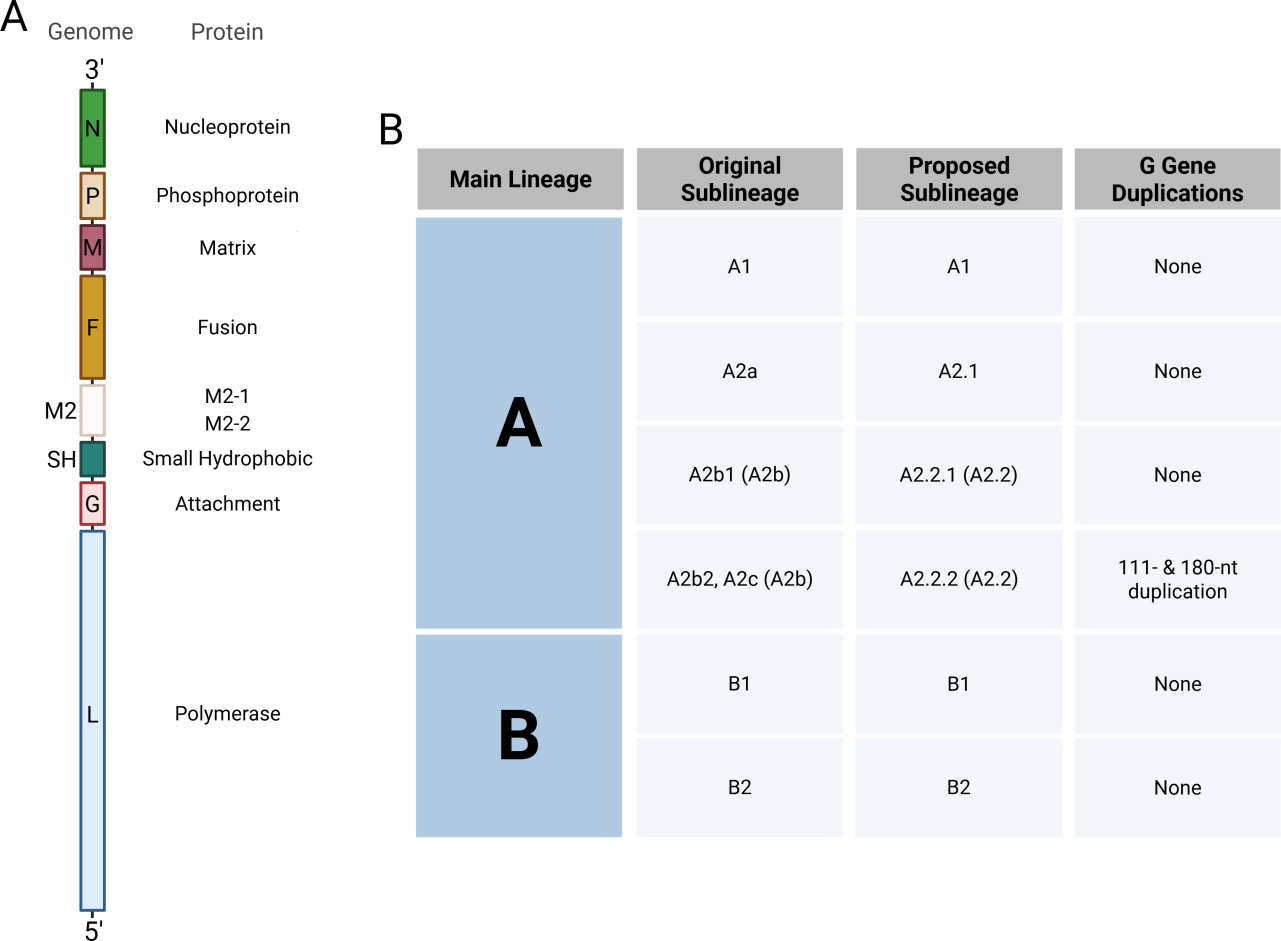
HMPV genome and genotypes. (**A**) Organization of the HMPV genome with the encoding proteins. (**B**) Classification of HMPV genotypes, lineages, and viruses with G gene duplications, and the original and proposed nomenclature for individual sublineages.

HMPV is a non-segmented negative-sense RNA virus in the *Pneumoviridae* family containing eight genes, totaling ~13.3 kb, that code for nine proteins. The produced proteins can be divided into four groups: glycoproteins, polymerase complex, structural, and M2 proteins. The glycoproteins consist of the fusion (F), attachment (G), and small hydrophobic (SH) proteins, which are all transmembrane proteins. F plays a role in attachment and fusion of the virus to cells (7, 8). The G and SH proteins have other functions discussed later in this review (9, 10). The nucleoprotein (N), phosphoprotein (P), and large polymerase (L) are the main constituents in the polymerase complex and are involved in viral replication and transcription (11–13). The matrix (M) protein is a structural protein that is involved in the assembly and budding of virus (14, 15). Lastly, M2-1/M2-2 are proteins that play various different roles in an HMPV infection, such as regulating viral RNA (vRNA) synthesis and inhibiting the immune system, though the role of M2-2 in immune antagonism has been disputed (16–20). These protein genes are organized in the following order: 3′-N-P-M-F-M2-SH-G-L-5′ (Fig. 1A).

The function of a number of the HMPV proteins was first extrapolated from studies of other viruses in the *Paramyxoviridae* and *Pneumoviridae* families, with human respiratory syncytial virus (RSV), also a member of the *Pneumoviridae* family, being most extensively studied. RSV and HMPV share homologous proteins, but RSV has two additional non-structural proteins (NS1/NS2) (21). Though it is often assumed that the proteins shared by RSV and HMPV have similar functions, there might be some divergence in their roles. In this review, we will focus specifically on the HMPV proteins and their functions.

## CLINICAL PATHOLOGY AND STRAINS

### Clinical pathology and prevalence

In the initial study from 2001, van den Hoogen et al*.* examined samples from patients who displayed symptoms similar to RSV, but for which an infectious agent had not been identified. These patients were then determined to be infected with HMPV (1). This study, and other work, showed HMPV-infected patients displayed classical respiratory tract infection symptoms that ranged from mild respiratory problems, such as congestion, fever, and coughing, to severe complications like bronchiolitis, hypoxia, and pneumonia (4, 22–24). In 2003, a study examining pathology and prevalence confirmed that HMPV was the second most detected viral pathogen, with children and immunocompromised patients being the largest groups hospitalized with HMPV (4). Multiple other studies have compared the prevalence of HMPV with that of influenza, RSV, and rhinovirus. The general trend suggests that hospitalizations with influenza and rhinoviruses are the most common, followed by RSV and HMPV, though these incidences vary per study and test population (25–30).

A meta-analysis examining 75 previous studies estimated the prevalence of HMPV infections in patients admitted to the hospital with acute respiratory infections (ARIs) as being 6.24% (2). Interestingly, there was an increase in the incidence rate for studies conducted further from the equator. This corroborates seasonal data that suggests HMPV is more prevalent in winter months (31, 32). In 2018, Wang et al*.* estimated that there were 14.2 million HMPV-associated ARI in children under 5 years of age, with 64% of in-hospital HMPV-positive deaths occurring in infants younger than 6 months (33). However, elderly individuals are also predisposed to developing severe ARIs. Kulkarni et al*.* estimated the global burden of HMPV in adults 65 years or older and approximated a total of 473,000 HMPV-associated hospitalizations in 2019 (34). Altogether, HMPV infections are seasonal, common, and continue to be a major health risk globally.

### Genotypes and emergence

Two different genetic groups were identified in the initial report of HMPV (1). Subsequent studies with more isolates revealed clustering of HMPV into two main genotypes (A and B) and four sublineages (A1, A2, B1, and B2) (35–38). Large-scale studies revealed ongoing virus evolution, particularly within the A2 lineage, which led to new sublineages over time labeled A2a (A2.1), A2b (A2.2), and A2c (A2.2.2) (39–41). Additional studies suggested that the A2b lineage should be further divided into A2b1 (A2.2.1) and A2b2 (A2.2.2), and that A2b2 can be mistaken for lineage A2c (42–45). These confounding inconsistencies in genotype nomenclature highlight the need for a unified genotyping and nomenclature system, similar to what has been proposed for RSV (46). For both HMPV and RSV, clear criteria have been suggested for genotype classification, including the adaptation to a numeric naming system, as is common for influenza viruses (47, 48). Going forward, this review will use the proposed nomenclature for genotypes and sublineages as shown in Fig. 1B.

Five recent studies representing variations in population sampling, years of study, regions, and detection methods were analyzed (Table 1) (41, 47, 49–51). These studies specifically investigated hospitalized patients presenting with ARI, but the detection methods varied, including which and how many genes were analyzed for genotyping. Together, these studies did not detect the circulation of a dominant genotype. Though they showed limited circulation of some genotypes, like A1, which was only detected twice and not seen after 2012 in these studies. Additionally, the analysis of these longitudinal studies shows that any genotype can circulate in any given year (41, 47, 49–51). For example, Jagušić et al*.* showed that B2 was the most abundant in 2011 and 2012, but B1 was in 2013 and 2014 (41). Ye et al*.* observed that B1, B2, and A2.2.2 were the most common genotypes in at least 1 year of the study (50). Together, these data suggest that various HMPV genotypes can circulate at the same time, with one of them often dominating in a particular year. However, the general trend suggests that genotypes B1, B2, and A2.2 (A2.2.1 and A2.2.2) tend to be identified more often.

**TABLE 1:** Frequency and prevalence of HMPV genotypes^*a*^

Study	Total specimens (*n*)	Years of study	Region	Genotype count (*n*)					
				B1	B2	A1	A2.1	A2.2^*b*^	
								A2.2.1	A2.2.2
Jagusic et al. (41)	80	2011–2014	Croatia	12	30	0	11	27	
Zhu et al. (49)	290	2010–2016	China	68	82	1	0	139	
Ye et al. (50)	54	2010–2019	China	27	9	0	0	18	
Otomaru et al. (51)	195	2007–2017	Vietnam	37	19	0	0	139	
Groen et al. (47)	130	2005–2021	Netherlands	14	35	1	15	21	44
Total	749			158	175	2	26	388	

^*a*^ Data from five studies are shown with the total number of specimens (*n*), years of study, region, and the number of isolates from each genotype detected. If a genotype was not listed, it was assigned a “0”.

^*b*^ If A2.2.1 and A2.2.2 genotypes were not defined, or A2.2 was counted alongside A2.2.1/A2.2.2, then they were counted as the A2.2 genotype.

Recent studies reported on strains containing a 180-nucleotide duplication in the G gene (43, 52), and Saikusa et al. reported a 111-nucleotide duplication (44, 53). Viruses with these duplications have been shown to circulate worldwide, and all belong to the A2.2.2 lineage (Fig. 1B) (42, 47). One study suggested that adults infected with A2.2.2 strains containing the G gene duplications were 3.45× more likely to develop lower respiratory tract infections when compared to A2.2.2 strains not containing a duplication in the G gene (54), suggesting a potential evolutionary advantage for these G gene duplications. Therefore, large-scale surveillance studies are needed to elucidate the impact of these duplications. Leyrat et al*.* suggested that the G protein is heavily glycosylated, and the extracellular region is intrinsically disordered (55). Piñana et al. hypothesized that this disordered region helps the G protein shield the F protein from the host immune response, and that the additional amino acids encoded by the 111-nucleotide and 180-nucleotide segments further conceal the antigenic sites on the F protein (54). However, this hypothesis remains to be validated experimentally.

## ENTRY AND FUSION

### Attachment and entry

Most *paramyxoviruses* and *pneumoviruses,* including HMPV, have two main surface glycoproteins known as the F and G proteins, though some viruses have a hemagglutinin-neuraminidase and hemagglutinin (H) instead of a G protein (56–61). HMPV, like a small number of *paramyxoviruses* and *pneumoviruses,* also contains a SH protein that may serve as a viroporin (1, 10, 62). Inoculation with HMPV lacking the SH protein (rHMPV-ΔSH) resulted in titers only marginally less compared to inoculation with wild-type (WT) HMPV (63). In a subsequent study, rHMPV-ΔSH showed a difference in infectivity depending on the specific strain used, but all strains produced infectious virus, further demonstrating that the SH protein is dispensable in infections (64). The G protein has been identified as the key receptor binding protein for many *paramyxoviruses*, though the HMPV G protein is dispensable for infections in cell culture and animal models (16, 56, 63). Infection in human airway epithelial cells showed that inoculation with a recombinant virus lacking the G protein (rHMPV-ΔG) led to significantly lower replication compared to WT HMPV, although there was a difference in attenuation based on the HMPV strain used (64). Experimental infections of non-human primates with HMPV showed that rHMPV-ΔG infection resulted in 6-fold and 3,200-fold lower infectious titers compared to inoculation with WT virus in the upper and lower respiratory tract, respectively (63). This demonstrates that the G protein is involved in viral spread in 3D cultures and animal models, which may be through increased viral budding, evading the immune system, or expanding cell tropism. Additionally, these data show that the HMPV G protein is not essential for attachment in 2D cultures. As discussed below, the F protein has been shown to have attachment-like properties.

**Fig 2:**
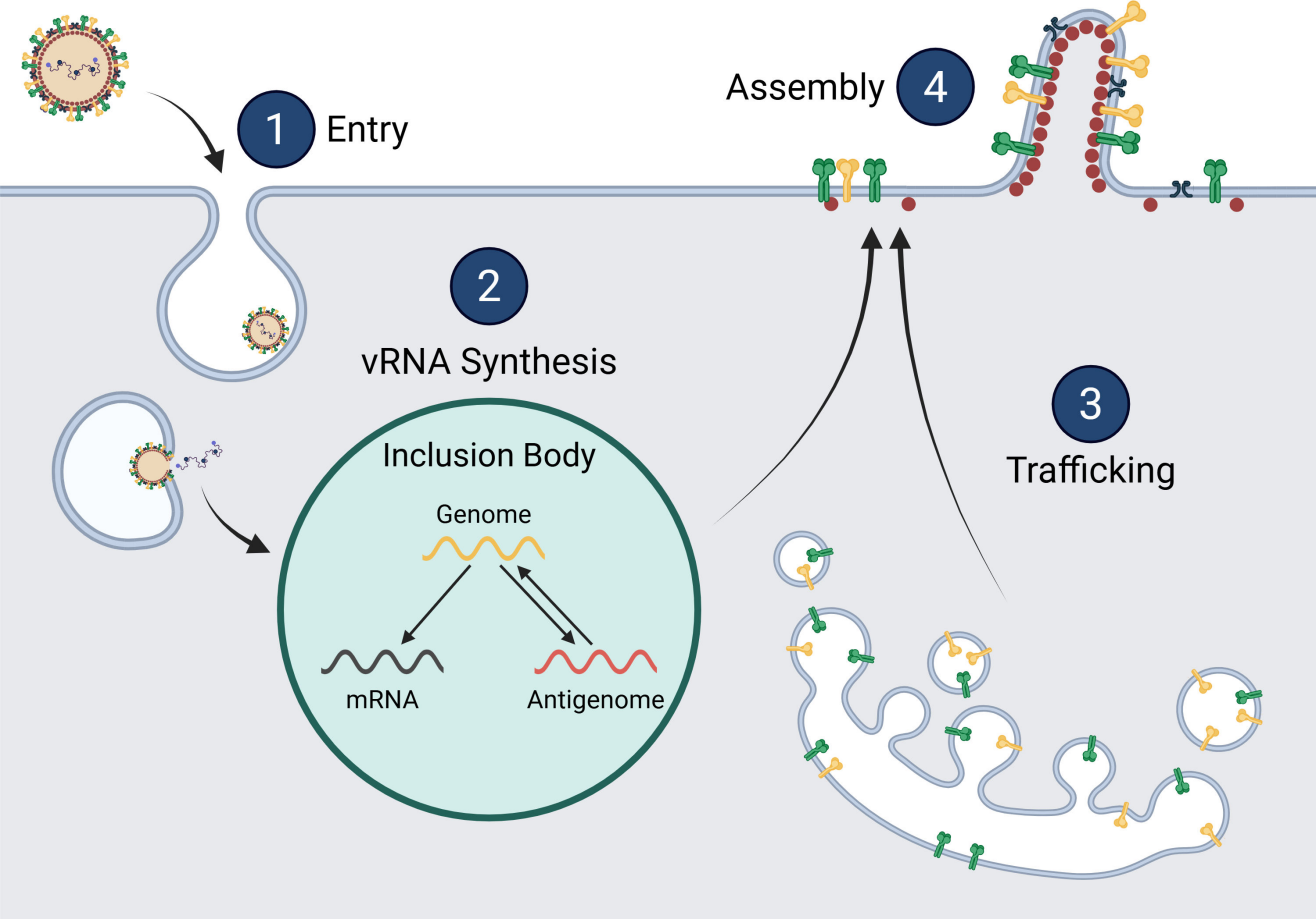
HMPV lifecycle. HMPV infections begin with (1) virus attachment and entry. Once the cell is infected, the virus (2) replicates its viral genome (yellow/red) and transcribes viral genes (gray) within inclusion bodies. Proteins such as G (green), F (yellow), SH (blue), and M (red) are synthesized and (3) trafficked through the cell to the plasma membrane. Proteins localize in specific areas and start the (4) assembly of viral particles through the concerted action of many viral proteins.

The F proteins of *pneumoviruses* are trimeric class I fusion proteins that are initially in a metastable pre-fusion state. Upon host cell binding or environmental stimuli, F is triggered to fold into its post-fusion state through a set of conformational changes that include exposure of the fusion peptide, its insertion into the opposing cell membrane, and subsequent re-folding of F to a form which brings the target and viral membranes into close proximity. The energy released by these conformational changes leads to lipid mixing and pore formation, which allows the transfer of the viral nucleocapsid into the host cell cytoplasm (Fig. 2, step 1). The F protein is produced as an inactive trimer, but becomes functional when cleaved by proteases prior to entry. It has been shown that TMPRSS2 and other biologically relevant proteases actively cleave the HMPV F protein, and that expression of TMPRSS2 in cell lines can lead to successful propagation (8, 65–67). However, exogenous trypsin can substitute for these natural proteases.

Since HMPV G is dispensable in cell culture, it was hypothesized that the HMPV F protein is involved in both attachment and fusion (68). The F protein was shown to interact with heparan sulfate (HS) proteoglycan, an extracellular glycan peptide that is commonly expressed on the surface of many different cell types (68, 69). Cultured cell lines that do not express HS proteoglycans showed a near 90% decrease in HMPV infection, regardless of whether WT or rHMPV-ΔG viruses were used (68). Other studies have further defined the specific moieties on glycans that interact with the HMPV F protein (70) and have shown that the addition of K5 polysaccharide derivatives, mimicking HS, can block viral infection in monolayer and 3D human airway epithelial cultures (71). In addition, attachment has been suggested to involve an interaction between the Arg-Gly-Asp (RGD) motif in the F protein and RGD-binding integrins (72). More studies have shown that HMPV infectivity can be inhibited by antibodies targeting specific integrins (73), and that the G protein was not needed for this interaction (74, 75). Live-cell imaging further demonstrated that HMPV enters the host cell through clathrin-mediated endocytosis, with subsequent fusion occurring within endosomes (76). Some strains of HMPV utilize a pH-dependent triggering mechanism for the conformational change of the F protein, further suggesting that entry occurs through endocytosis to allow the viral particle access to the low pH compartments of endosomes (8, 76). F residues G294 and H435 have been associated with pH-dependent triggering of the F protein (7, 77–79). However, the exact mechanisms of how the F protein interacts with HS, other glycans, and integrins to facilitate entry need further investigation.

## REPLICATION AND TRANSCRIPTION

### Viral RNA synthesis

**Fig 3:**
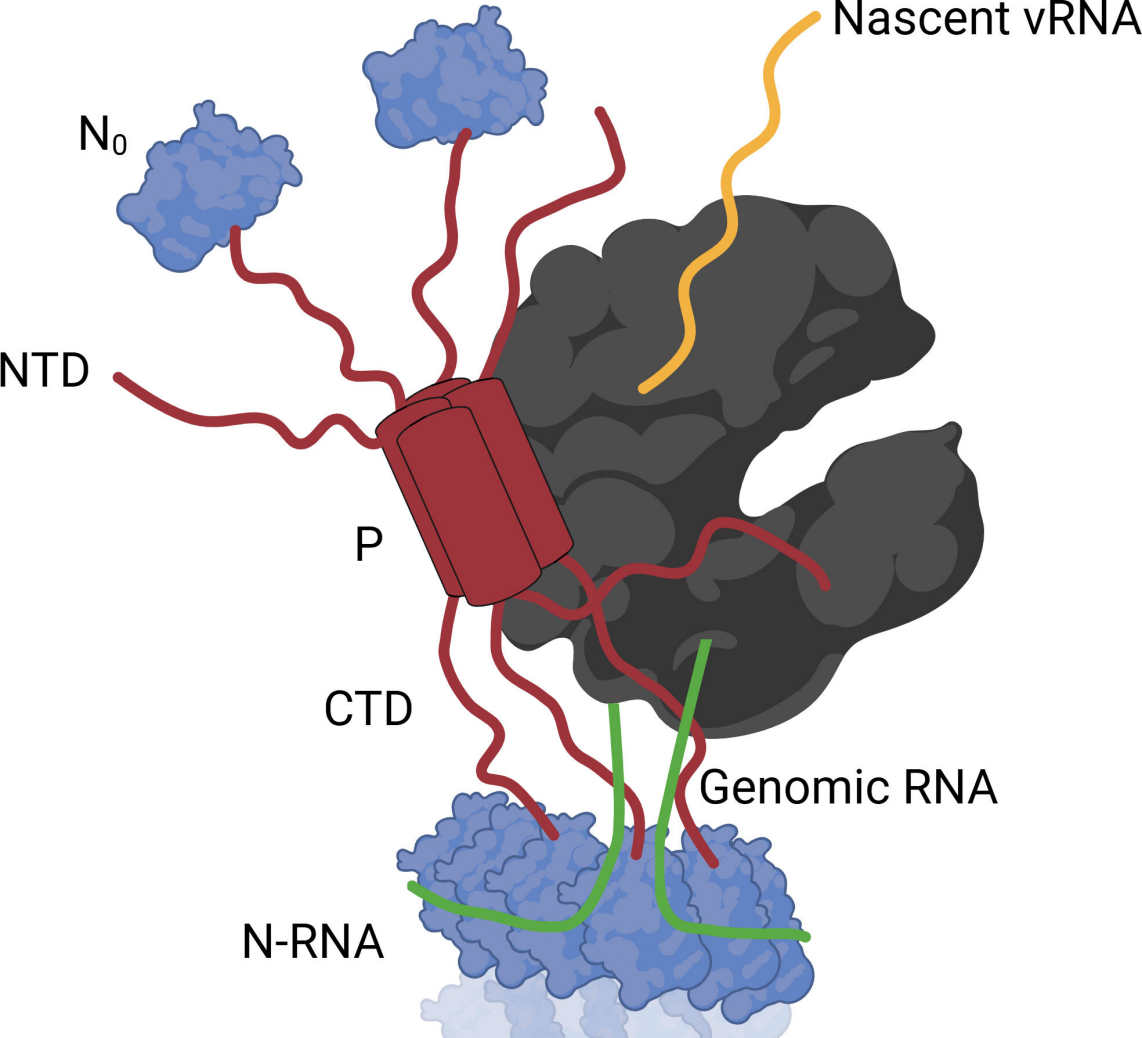
Proposed HMPV polymerase complex organization. The HMPV polymerase complex consists of three proteins: N (blue), P (red), and L (gray). Genomic RNA (green) is encapsidated by N in an oligomeric structure known as N-RNA. During replication and transcription, N-RNA is kept in close proximity to L through interactions with the C-terminal domain (CTD) of P. As new RNA (yellow) is being synthesized, free N (N_0_) can encapsidate the RNA through interactions with the N-terminal domain (NTD) of P.

HMPV is a negative-sense RNA virus, meaning that the packaged HMPV genome cannot serve as mRNA, but instead must be both transcribed to viral mRNA (vmRNA) and replicated to form anti-genomic RNA (Fig. 2, step 2). The antigenome then serves as a template for the synthesis of the viral genomic RNA. In contrast to RSV, limited studies have been reported on HMPV viral RNA synthesis, so RSV currently serves as a critical comparative model. Studies with RSV have shown that successful vRNA synthesis requires four proteins: N, P, L, and M2-1 (80), whereas the M2-1 protein is not essential for HMPV infection (Fig. 3) (18, 81). To initiate either replication or transcription, the RSV L protein binds to the leader (*le*) region at the 3′ end of the genome (81–83). *Pneumoviral* replication begins at the first (+1) position, where adenosine triphosphate (ATP) is used to complement uracil at the 3′ end of the genome (84–87). Then, the first step of replication occurs and produces the antigenome, which is then used as a template to synthesize additional genomes. As with many negative-sense RNA viruses, HMPV and RSV have trailer (*tr*) regions at the 5′ end of the genome (81, 83, 87, 88). This *tr* region serves as the initiation site for the L protein at the 3′ end of the antigenome. The *le* and *tr* regions are critical for viruses in the *Mononegavirales* order, and many mutations in these regions result in a loss of replication (88, 89).

Negative-sense RNA viruses, like HMPV, initiate transcription and replication on the same *le* region in the genome, so there is likely a mechanism to regulate vRNA synthesis. Though it has not been shown for HMPV to date, in RSV, ATP and guanosine triphosphate (GTP) concentrations play a role in balancing replication and transcription (84, 87). The balance between replication and transcription for some viruses from the *Mononegavirales* order has been shown to depend on increasing N and C protein (a second protein encoded in the P gene) levels (85, 90–94). However, *pneumoviruses* like HMPV and RSV do not express a C protein. Another mechanism for balancing replication and transcription is through dimerization of the polymerase, which has been suggested for human parainfluenza virus type 3 and potentially for other members of the *Mononegavirales* order (92–94). The role of these or other mechanisms for regulating the balance between transcription and replication for HMPV remains to be determined.

RSV transcription initiates at the +3 position, where GTP complements cytosine (95). During RSV transcription, when the polymerase complex reaches ~25 nucleotides into the genome, it releases the nascent RNA, scans for the first gene start (GS) sequence, and begins transcription of the first gene until it reaches the gene end (GE) sequence (85, 87). After a transcript has been produced, it is thought that the U-rich GE sequence leads to polyadenylation of the mRNA transcript (85, 96). Although it seems likely, whether HMPV vRNA synthesis occurs similarly to RSV needs to be investigated.

A key feature of negative-sense RNA viruses is the gradient level of gene expression, meaning that genes toward the beginning of the genome are transcribed the most, and downstream genes have increasingly fewer transcripts (97). This gradient is proposed to be due to partial dissociation of the L protein at GE signals or U-rich sequences, but there may also be roles for polycistronic mRNAs, N regulation, or changes in polyadenylation that affect RNA stability (97–102). The M2-1 protein of RSV has been described as a transcription anti-terminator, or elongation factor, causing read-through at GE signals during transcription (103, 104). A similar function has been proposed for the M2-1 protein of HMPV, but unlike the RSV M2-1 (105–107), HMPV M2-1 is not essential for the transcriptional process, although it is often included in rescue systems and vRNA synthesis assays (18, 81). Interestingly, rHMPV-ΔM2 mutants as well as individual ΔM2-1 and ΔM2-2 mutants only showed a slight reduction in infectious titers upon infection of Vero cells compared to WT virus (18). Another study has shown that rHMPV-ΔM2-2 virus stocks contained defective interfering RNAs and hypermutated genomes (16), and that the transcription profiles of ΔM2-2 mutants were different than that of the WT virus (20), consistent with other studies (18, 108). These studies demonstrated a potential role for M2-2 in regulating transcription, likely in concerted action with the M2-1 protein. This role for M2-2 has also been observed in RSV, showing that M2-2 may serve a similar role in both *pneumoviruses* (109, 110).

The structure of the HMPV L protein was elucidated in 2019 and demonstrated the presence of many conserved domains observed in other viral RNA-dependent polymerases (11). These include the functional domains: RNA-dependent RNA polymerase (RdRp), GDP polyribonucleotidyltransferase (PRNTase), and methyltransferase (MTase). The RdRP is responsible for adding free nucleotide triphosphates (NTP) onto the newly synthesized strand. The PRNTase domain for viruses in the *Mononegavirales* order is involved in capping pre-mRNA with an inverted guanosine diphosphate at the beginning of mRNA transcripts (111–114). The MTase domain is also involved in the maturation of transcripts by adding methyl groups to the 5′ cap of mRNAs (115–117). These regions work together to synthesize new mRNAs. The polymerase complex (schematically shown in Fig. 3) presents four monomers of P interacting through their oligomerization domain (OD), with one monomer extending its C-terminal domain around L (Fig. 3) (11). The N-terminus and C-terminus of P are intrinsically disordered, presenting significant flexibility for P interactions with other proteins. It is hypothesized that the N-terminal and C-terminal domains of P that do not interact with L can interact with free N (N_0_) and N-RNA, respectively (11–13, 118–120). For RSV, it was shown that the M2-1 protein also interacts with the region between residues 100 and 120 of P, suggesting it to be an important regulatory factor, though this has not been shown for HMPV (121, 122). Research to date shows that there are similarities in the replication process for viruses within the *Pneumoviridae* family, but there are also differences, such as M2-1 not being necessary for HMPV vRNA synthesis. Therefore, more research is needed to examine the differences between *pneumoviruses* and the role for M2-1 in HMPV.

### Inclusion bodies

HMPV, like other viruses in the *Mononegavirales* order, forms cytoplasmic liquid-liquid phase-separated regions called inclusion bodies (IBs), also known as replicative centers or viral factories (Fig. 2, step 2) (123–128). The minimal components needed to form intracellular HMPV IB-like structures are the N and P proteins, which is similar to many other viruses such as RSV, measles virus, and rabies virus (129–133). However, for HMPV, it has been shown that the P protein alone forms phase-separated regions in purified systems (134). The N protein is an RNA binding protein that encapsidates RNA, resulting in a coil-like structure as N oligomerizes along RNA. One complete turn of the nucleocapsid typically involves 10 Ns, though 9-mers, 11-mers, and 12-mers have been observed, with each monomer occupying seven RNA nucleotides (12, 135). Free monomeric N (N_0_) is also present and is critical for encapsidating newly synthesized viral genomic RNA. It has been shown that the C-terminal six amino acids of the HMPV P protein are necessary for binding to N-RNA complexes (Fig. 3) (12, 118). Deletion of the C-terminal domain of the P protein prevented IB formation, while the deletion of the N-terminus and OD did not, demonstrating that N and P play a role in IB formation (13, 118). Phosphomimetic or phospho-dead mutants change the diffusion rate of IBs, demonstrating that the P protein phosphorylation can modulate interactions that contribute to mobility within IBs (13).

It is hypothesized that IBs function to increase the local concentration of vRNA and proteins involved in replication and transcription, facilitating enhanced RNA synthesis. vRNA has been shown to be present in HMPV IBs through fluorescence *in situ* hybridization, while studies with related viruses, such as RSV, have utilized 5′ ethynyl-uridine labeling to show that RNAs are actively synthesized in IBs (127, 136, 137).

## VIRUS ASSEMBLY, TRAFFICKING, AND CELL-CELL SPREAD

### Virus assembly and budding

**Fig 4:**
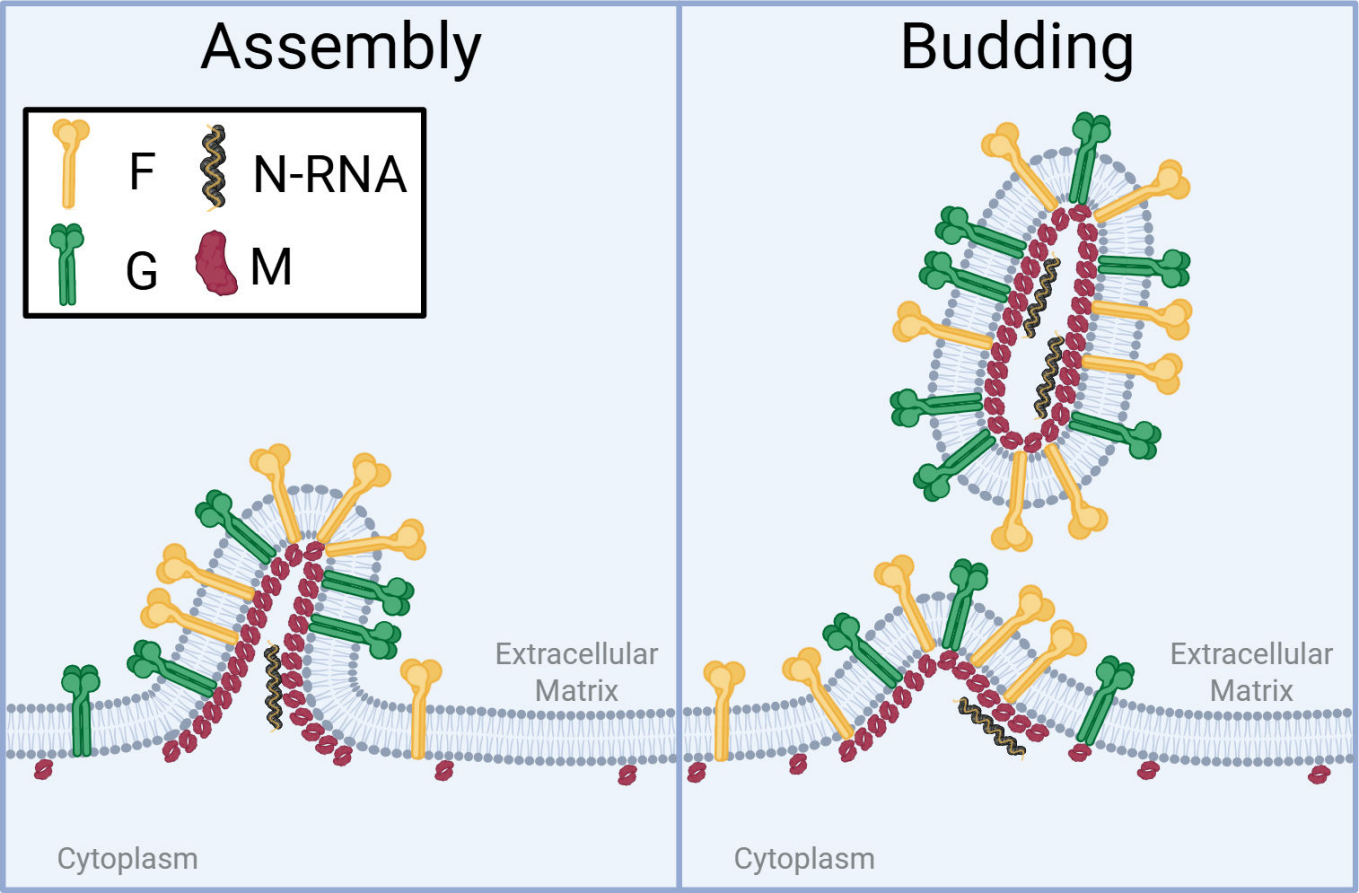
Schematic of HMPV assembly and budding. Viral assembly occurs at the plasma membrane of infected cells. M (red), F (yellow), G (green), and N-RNA (yellow/black) accumulate at specific areas on the plasma membrane and initiate the assembly process. Budding viral filaments are formed as more proteins localize at assembly sites. The release of the virions results in free viral particles, though many remain cell-associated with HMPV.

Virus assembly and budding involve gathering viral components at the host cell membrane and then pinching off to release infectious particles (Fig. 4). HMPV assembly has not been extensively studied; as such, the evaluation of related viruses is an important starting point (Fig. 2, step 4). For all negative-strand RNA viruses studied to date, the M protein is a key facilitator of the assembly process, with demonstrated interactions with critical viral and cellular factors. To produce virus, HMPV forms virus-induced filaments at the plasma membrane of infected cells which localize with many viral proteins and the nucleocapsid and are shed as free viral particles. *Pneumovirus* M has been implicated in filament formation, since rRSV-ΔM produced only smaller filaments that are likely not functional virus (138). This suggests that M is necessary for the maturation of filaments during assembly, which would ultimately lead to the formation of new viral particles. However, the F protein likely also plays a role, as it has also been shown that the C-terminal domain of the RSV F protein is involved in filament formation and recruitment of the N, M, and P proteins into virus-like particles (VLPs), which are particles that resemble viruses (139, 140). Since the RSV M and F proteins have both been associated with filament formation, they may be interacting partners to recruit other viral proteins to assembly sites. In contrast to RSV, the crystal structure of the HMPV M protein dimer showed a unique calcium-binding site (14). While a specific role for calcium in assembly and budding has yet to be established, this finding suggests that calcium may regulate HMPV M interactions (141). Overall, the HMPV M protein appears to be involved in virion assembly; however, the mechanisms that control this process are not well understood.

After assembly, budding occurs and viral particles are released (Fig. 4). Many HMPV particles remain cell-associated, rather than releasing into the cellular environment. Some *paramyxoviruses*, such as Nipah virus, utilize endosomal sorting complex required for transport (ESCRT) to promote virus release from infected cells (142). In HMPV, ESCRT inhibition does not impact viral particle release, so ESCRT does not seem to be involved (143). For many negative-sense RNA viruses, the M protein is a key factor for budding (15, 144–148). However, different expression levels of the M, F, and G proteins may change how efficiently the virus buds, overall shape, and/or the infectivity of each virion (149). The HMPV G and M proteins have been shown to form VLPs alone, indicating that one or both proteins may be involved in budding (150). It has been reported that when the P, M, and F proteins come together, they are capable of budding filamentous RSV virions, and F protein C-termini are needed (151). Interestingly, the RSV M2-1 protein with the M and F proteins has also yielded immunogenic VLPs, suggesting various combinations of proteins can yield VLPs that mimic infectious virus (152). However, more work is needed to understand how the HMPV proteins and the cellular factors aid in budding.

Multiple studies of other viruses have implicated interactions with cellular lipids in assembly and budding. Recently, it was shown that the Nipah and measles virus M proteins interact with phosphatidylinositol 4,5-bisphosphate (PIP_2_) and phosphatidylserine (PS), although PS did not induce membrane deformation at low protein densities (153). The Nipah M protein contains basic patches that serve as binding pockets that interact with PIP_2_. This alters the M-dimer conformation from a concave to a flat surface, induces membrane deformation, and leads to viral budding when enough M is present. Mutations to the PIP_2_ binding site or dimer interface disrupted viral budding, showing that both functions are important. RSV has been shown to bind PS rather than PIP_2_ (154). To date, HMPV M has not been directly shown to interact with either PIP_2_, PS, or other lipids.

### Trafficking

Viral genomes and proteins are synthesized in the cytoplasm, so trafficking is a necessary process to efficiently assemble and bud virus. The HMPV F protein is a type I transmembrane protein that contains a signal peptide at its N-terminus (155), while the HMPV G and SH proteins are type II glycoproteins. (Fig. 2, step 3). These glycoproteins are synthesized in the endoplasmic reticulum and traffic through the secretory pathway to the plasma membrane. Although, it has been shown that a significant portion of the F protein population is not present on the plasma membrane of the cell (8). This is potentially due to longer residence in the secretory pathway or internalization via the endocytic pathway after arrival at the plasma membrane.

Currently, only a few studies have examined the transport of vRNA, and none have focused on the trafficking of HMPV vRNA and the associated polymerase complex. However, studies on RSV and other negative-sense RNA viruses have provided insight into potential mechanisms of transport. The cellular cytoskeleton, including both actin and microtubules, is thought to play a major role in the transport of viral components (156, 157). The HMPV P protein has been shown to localize with actin and induces the formation of membrane extensions (158). The RSV P and M proteins have also been shown to interact with tropomyosin, an important protein in actin organization (159). The RSV M protein has also been demonstrated to interact with actin and palladin, suggesting that it may bridge the cytoskeleton and viral ribonucleoprotein complexes for trafficking (160–164). Investigations on the RSV M protein have shown interactions with P, and potentially N and F, supporting the hypothesis that M may recruit many viral proteins to assembly sites (138, 165, 166). Therefore, it is reasonable to hypothesize that the *pneumoviral* M and P proteins function together to drive changes in actin architecture, vRNA localization, and filament formation (164, 167, 168), but further studies are needed to elucidate this process.

Microtubules may also facilitate vRNA transport to the plasma membrane since tubulin was found to colocalize in viral filaments (158). Additionally, it is suggested that the RSV G protein and intracellular vesicles could play a role in vRNA transport (169), as live cell imaging showed that RSV genomic RNA traffics along microtubules (170, 171). This suggests that vRNA, G, and other viral proteins may assemble prior to reaching the membrane (169). Moreover, it has been shown that RSV ribonucleoprotein complexes colocalize with Rab11, a GTPase involved in the recycling endosome (170). However, for HMPV, it has been shown that RNA trafficking does not depend on Rab11 or tubulin (127). These findings highlight the need to further investigate viral trafficking of HMPV components.

### Direct cell-cell spread

**Fig 5:**
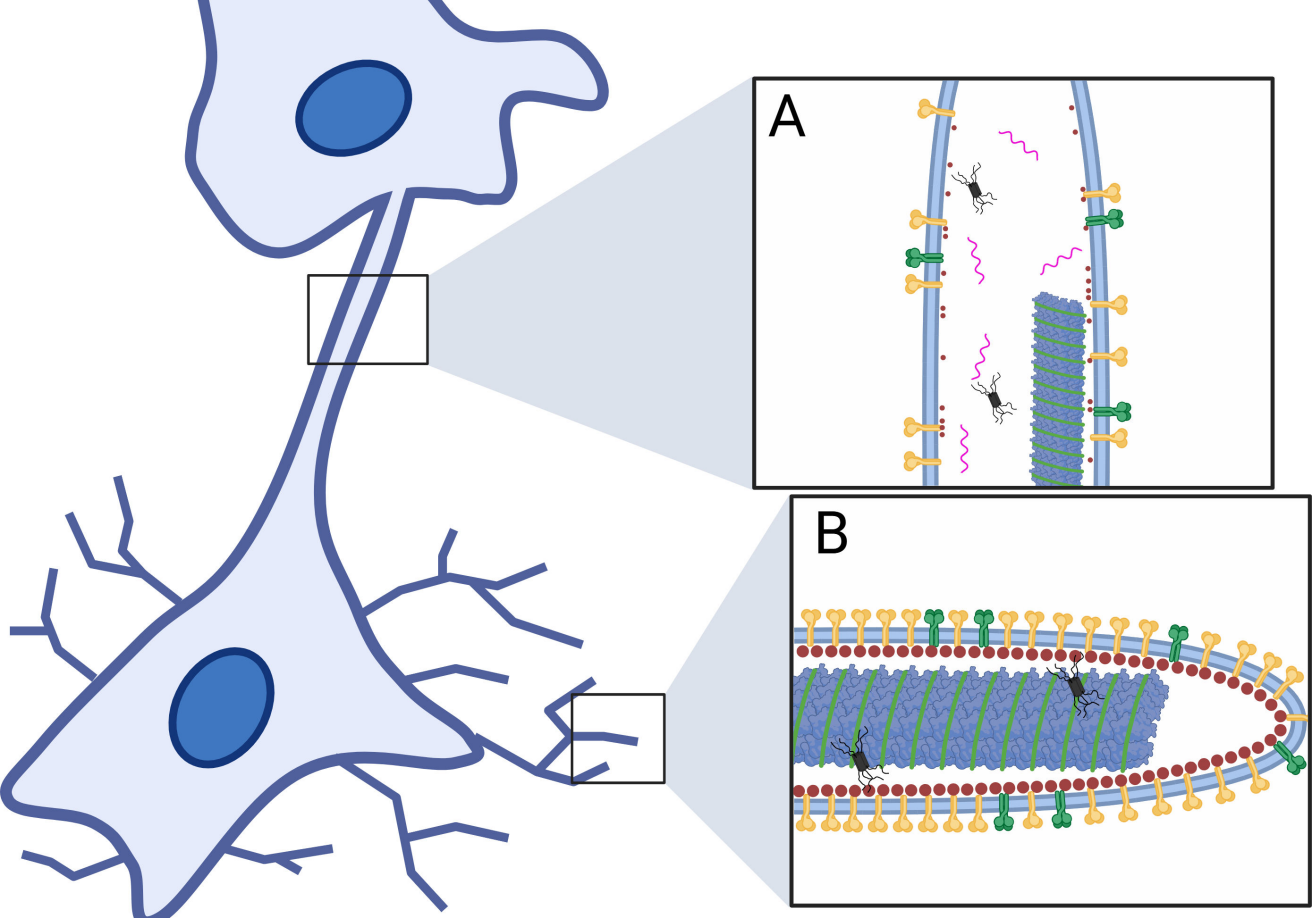
Organization of HMPV filaments and intercellular extensions. HMPV-infected cells can form viral filaments and intercellular extensions. (**A**) Extensions are larger structures that extend from the cell body to contact a neighboring cell. They can contain many viral proteins such as P (black), M (red), N (blue), F (yellow), G (dark green), genomic vRNA (light green), and positive-sense vRNAs (pink). (**B**) Filaments are smaller structures that have high concentrations of F (yellow) and genomic RNA (light green). Though, filaments also contain G (dark green), M (red), P (black), and N (blue).

The budding virus of HMPV appears as filaments emerging from the cell body and contains almost exclusively negative-sense viral RNA (Fig. 5) (158, 172). However, HMPV can also promote direct cell-to-cell spread through intercellular extensions that form during infection (172, 173), which contain positive-sense and negative-sense vRNAs (Fig. 5) (158, 172). These structures have properties similar to nanotubes and can allow dye transfer between two connected cells (172, 174, 175).

To better understand what leads to intercellular extension formation, membrane structure changes were observed when individual HMPV proteins were expressed through transient transfection. HMPV M, F, and P were all observed to induce changes in membrane structures (158). In this study, the N, M, and P proteins were also shown to be present in extensions in HMPV-infected cells. Moreover, proximity ligation assays, a fluorescent microscopy assay used to identify close proximity of two proteins, showed that the P protein and actin were near each other in infected cells (176). In addition, the incorporation of cytochalasin D in this assay resulted in a reduction in the number of extensions, confirming that these structures are actin dependent (158). For RSV, other viral proteins, such as the M protein, have been shown to interact with actin and actin-related proteins, suggesting that the HMPV M protein might have similar roles (160, 161). Together, the formation of extensions might be due to the localization of one or more HMPV proteins that can interact with actin-related proteins.

In addition to evaluating individual viral proteins, live cell imaging of HMPV-infected cells confirmed that IBs can transfer through intercellular extensions to neighboring cells (172), supporting a novel mechanism of cell-cell spread that would allow transfer of many viral genomes, along with the replication machinery, at one time. Future studies are needed to clarify how these structures form and what role they have in infection and possibly in evasion of the immune system.

## EVADING THE ANTIVIRAL RESPONSE

### Innate immune antagonism

Viral infections typically induce the cellular innate immune response, which facilitates changes to resist viral infections. HMPV, like most viruses, has evolved specific mechanisms to evade the innate immune response. To date, G, SH, and M2-2 have been reported to be interferon antagonists, though recent research suggests that these proteins do not inhibit the innate immune response. Therefore, it is not clear which HMPV proteins aid in evading the host response.

In a normal infection, RNA sensors in the cytoplasm, such as retinoic acid-induced gene (RIG)-I, detect viral RNA and initiate an innate immune response. The HMPV G protein has been suggested to interact with RIG-I and to inhibit interferon (IFN)-β promoter expression (177, 178), but also to play a role in evading toll-like receptor 4-dependent signaling (179, 180). However, another study using G-targeting silencing RNAs to knock down G expression suggested that the G protein is not a significant type I interferon antagonist (181). In addition to the G protein, the SH protein has been associated with inhibiting IFN gene transcription in dendritic cells, which was shown through an increase in type I IFNs in rHMPV-ΔSH-infected cells (182). Others have shown SH-mediated IFN inhibition by examining STAT1 (signal transducer and activator of transcription-1) activation (183), interleukin-6 (184), and NF-κB activity (179, 185). However, de Graaf et al*.*, using microarrays and mass spectrometry-based techniques, concluded that there were only minor changes in transcript or protein expression levels in rHMPV-ΔSH-infected cells when compared to WT HMPV (186). In addition, Groen et al. also demonstrated that rHMPV-ΔG and ΔSH viruses did not activate the IFN response when compared to WT virus (16). They have shown that HMPV accumulates defective interfering RNAs (DIs) rapidly when virus stocks are generated upon multiple passages with a high multiplicity of infection, which could explain the contradicting data.

The M2-2 protein has also been suggested to serve as an interferon antagonist (19, 187, 188). Ren et al*.* showed that the M2-2 protein co-immunoprecipitated with mitochondrial antiviral-signaling protein, a key protein in the innate immune response (19, 189). These studies also showed that infection with rHMPV-ΔM2-2, or with viruses with mutated PDZ motifs in the M2-2 protein, led to increased cytokine and chemokine secretion compared to WT virus (189, 190). However, Groen et al. showed that although infection with rHMPV-ΔM2-2 led to activation of the IFN pathway, the viral genomes of these viruses were hypermutated and virus stocks contained high amounts of DIs, which are known to be strong activators of the IFN pathway (16), suggesting the effect of M2-2 on the innate immune response is indirect. A subsequent study demonstrated that a chimeric RSV expressing the HMPV M2-2 protein in place of the non-structural proteins 1 and 2 (NS1/NS2), the IFN antagonists of RSV, did not reduce IFN responses induced by RSVΔNS1/2, indicating that the HMPV M2-2 protein does not act as a robust IFN antagonist (20). This study suggested a role for the M2-2 protein during transcription and that its expression inhibits the production of DIs and thereby activation of the IFN pathway. Therefore, it remains to be elucidated how HMPV antagonizes the innate immune response.

### Inhibiting the adaptive immune system

The host adaptive immune response to HMPV is also critical to pathogenesis. The human adaptive immune response includes the recruitment of immune cells to identify infected cells and target them for cell death. This can be achieved through the production of antibodies that recognize viral proteins on the surface of infected cells, or through the expression of cellular proteins that alert the immune system that cells are infected. To examine these responses, HMPV infection has been studied in several animal models, including mice, cotton rats, ferrets, hamsters, and non-human primates (17, 191–194). Mice have been reported to sustain HMPV infections (195); however, some reports show that they are poorly susceptible (196). Additionally, immunocompetent mice are poorly re-infected with HMPV (24, 197, 198), which differs from humans, where HMPV re-infection occurs throughout life (199). In studies where mice were infected with HMPV, the animals developed similar clinical symptoms to infected humans, including showing increasing severity with age (200). Other animal models, such as cotton rats, hamsters, and ferrets, do not present observable clinical symptoms upon HMPV infection (24, 192). Experimental infections of non-human primates have shown that re-infection occurs in the presence of existing antibodies, but also that vaccines are protective against re-infection (63, 193).

The majority of studies of the HMPV immune response have been conducted with mice, where peak virus titers occur at ~5 days post-infection (dpi) (201, 202). The adaptive immune system includes T-cells that can recognize HMPV-infected cells and target them for cell death. Kolli et al*.* showed that lymphocytes from BALB/c mice, which fight infections through effector functions such as lysis and cytokine secretion, were also responsive to HMPV (CAN 97-83) with peak cell levels at 6 and 10 dpi for CD4^+^ and CD8^+^ lymphocytes, respectively (198). They also showed that antibody-targeted depletion of CD4^+^ lymphocytes resulted in a significant decrease in lung pathology score but resulted in similar HMPV titers when compared to negative controls. Interestingly, the depletion of both CD4^+^ and CD8^+^ lymphocytes leads to reduced inflammation and weight loss, but a significant increase in lung titers when compared to the individually depleted samples (198). These results demonstrate that CD4^+^ and CD8^+^ lymphocytes contribute to disease severity and HMPV replication. Importantly, it has been noted that HMPV (TN/94-49) infection in C57BL/6 mice hinders a proper CD8^+^ T cell response through prolonged expression of programmed death-1 (PD-1) (202–205). This inhibitory receptor dampens CD8^+^ lymphocyte function and impairs long-lasting immunological memory (206). This effect on PD-1 expression was seen across four main HMPV sublineages in infected C57BL/6 mice, though HMPV A1, B1, and B2 genotypes had slightly higher expression of PD-1 than the A2 strain (207). In addition to T cell responses, some proinflammatory and regulatory cytokines were much lower in HMPV infections when compared to RSV; however, other immune factors showed the opposite trend (208–210). It was also reported that type I and type III IFN responses yield different pathologies in HMPV infections. Zhang et al*.* demonstrated that inhibiting type-I IFN responses in C57BL/6 mice led to little-to-no weight loss while having similar levels of replication (211). Conversely, a reduction in type-III IFNs by neutralizing antibody treatment did not rescue weight loss but did lead to an increase in viral replication (211). These data demonstrate an inverse relationship between type I and III IFN responses and how they contribute to clearing the virus or disease pathology.

Antibodies are also an important part of the adaptive immune system, and they typically target the F protein, as this is the major antigen that the immune system recognizes (9). Skiadopoulos et al*.* showed that the SH and G proteins do not induce high levels of neutralizing antibodies, confirming the F protein as the major immunogen (9). Recent studies have evaluated patient antibodies and their target regions and neutralizing ability to determine how the immune system targets the F protein (212, 213). Cumulatively, they showed that the majority of patient antibodies do not target the apex of the HMPV F trimer (212, 213), likely due to N-linked glycan shielding (214). This varies from the apex-targeting antibodies produced by a natural RSV infection (215). Instead of targeting the apex, many of the strong neutralizing antibodies toward the HMPV F protein tend to not discriminate between pre-fusion and post-fusion forms (212, 216), though some strong pre-fusion-specific antibodies have been detected (212, 213). However, Rappazzo et al*.* identified an antibody that did target the apex and it provided protection against HMPV challenge (216). Interestingly, their study showed that 72% of patient pre-fusion F-specific antibodies displayed weak-to-undetectable neutralization of HMPV (216). Therefore, the majority of the pre-fusion F antibodies may detect cryptic sites that are poorly accessible in the native HMPV F trimer (216). Together, these studies show the complexities of HMPV F neutralization.

Early work with rHMPV-ΔG as a potential vaccine candidate resulted in a sixfold reduction of virus and a delayed peak titer in the upper respiratory tract of non-human primates compared to WT virus (63). Velayutham et al*.* showed that the loss of G reduced clinical disease in BALB/c mice, led to more activated T cells, and to the production of neutralizing antibodies that protected mice against a second challenge (217). However, they showed a significant decrease in neutralizing antibody titer at 4 and 6 weeks after primary rHMPV-ΔG infection compared to WT HMPV. Primary rHMPV-ΔG infection led to a larger decrease in body weight upon challenge when compared to WT HMPV, which may be due to reduced antibody neutralization from G-shielding on the F protein. This could be due to the disordered regions or glycosylation moieties on the G protein, as previously discussed; however, this hypothesis still needs to be thoroughly examined. Altogether, the incorporation of the G protein into immunizations may better mimic WT infections and induce a more robust neutralizing antibody response.

## CONCLUSION

Since its discovery in 2001, HMPV has been shown to be a common respiratory virus that can cause severe respiratory tract infections in all age groups, but primarily in infants, older adults, and individuals with underlying disease. Though much knowledge has been obtained about HMPV, more research in multiple areas is clearly needed. To better understand the evolution of HMPV and the circulation of different genotypes worldwide, surveillance and subsequent sequencing of viral strains are crucial. Additionally, the mechanisms of attachment and entry have been characterized, but the role of different entry pathways remains less clear. The main function of the G protein remains unknown, though it appears to be important for viral spread in 3D cell culture and animal models. Viral replication is a key target for the development of antiviral therapies but has been only sparsely examined for HMPV. Assembly and budding mechanisms have been established for many negative-sense RNA viruses; however, for HMPV, limited studies have been reported on these processes. In addition, the unique cell-cell spread pathway identified for HMPV represents an intriguing mechanism, but the role of this in infections remains to be defined. Lastly, significant work has focused on how HMPV evades adaptive immunity. These studies have demonstrated how the immune system responds and combats HMPV infection, providing more insight into how to design potential vaccines. However, more research is needed on the exact mechanism HMPV uses to evade the innate immune responses. Altogether, HMPV is an important respiratory pathogen for which much research has been done, but much remains to be discovered.
